# Supplementary material for: The impact of access to financial services on mitigating COVID-19 mortality globally
Source: PLOS Glob Public Health. 2023 Mar 17;3(3):e0001137. doi: 10.1371/journal.pgph.0001137 (PMC10022804; doi:10.1371/journal.pgph.0001137)
Supplement: S1 Table — (DOCX) [file pgph.0001137.s001.docx]

**S1 Table. Descriptive Statistics for Model Variables**

| **Variable** | **Obs.** | **Mean** | **Std. Dev.** | **Min.** | **Max.** |
| --- | --- | --- | --- | --- | --- |
| **Dependent Variable** | | | | | |
| ln(COVID-19 death rate per million, to 9/30/21) | 142 | 5.77 | 1.87 | -0.21 | 8.7 |
| **Demographic & Socioeconomic Variables** | | | | | |
| Population aged 65 & older, 2019 (%) | 142 | 10.11 | 6.91 | 1.26 | 28.4 |
| Population aged 0–14, 2019 (%) | 142 | 26.11 | 10.39 | 12.3 | 49.5 |
| ln(Population density per sq. mile, 2019) | 142 | 4.34 | 1.40 | 0.75 | 9.03 |
| Population in urban areas, 2018 (%) | 139 | 61.81 | 22.13 | 16 | 100 |
| ln(Per capita income, 2019) | 141 | 9.50 | 1.17 | 6.72 | 11.7 |
| Income inequality, 2019 (Gini) | 142 | 38.08 | 7.79 | 25 | 64 |
| **Population Health Variables** | | | | | |
| ln(Mortality from indoor air pollution per 100K, 2016) | 139 | 4.05 | 0.99 | 1.95 | 5.78 |
| ln(Diabetes prevalence, %, 2019) | 138 | 1.85 | 0.49 | 0 | 2.99 |
| ln(Lung cancer prevalence per 100K, 2018) | 139 | 2.40 | 1.08 | -0.77 | 4.08 |
| Mean body mass index, 2016 (kg/m^2^) | 139 | 25.50 | 2.03 | 20.6 | 29.6 |
| Raised blood pressure prevalence, 2015 (%) | 140 | 24.35 | 4.83 | 11 | 33.4 |
| Tuberculosis vaccine coverage, 1989–2018 (%) | 142 | 61.89 | 31.31 | 0 | 99 |
| **Health Infrastructure Variables** | | | | | |
| ln(Nurses and midwives per 10K, 2010–2019) | 139 | 3.28 | 1.24 | -2.85 | 5.38 |
| Health services effective coverage (UHC index), 2019 | 141 | 63.79 | 16.42 | 22.3 | 96.34 |
| **World Bank Region Dummy Variables** | | | | | |
| East Asia & Pacific | 142 | 0.12 | 0.33 | 0 | 1 |
| Europe & Central Asia | 142 | 0.32 | 0.47 | 0 | 1 |
| South Asia | 142 | 0.04 | 0.20 | 0 | 1 |
| Middle East & North Africa | 142 | 0.11 | 0.32 | 0 | 1 |
| Sub-Saharan Africa | 142 | 0.25 | 0.43 | 0 | 1 |
| **Financial Access Indexes from Principal Components Analysis** | | | | | |
| Broad access to and use of formal finance index | 142 | 0 | 3.521 | -4.858 | 9.056 |
| Reliance on alternative, informal,44 & distress finance index | 142 | 0 | 1.391 | -2.342 | 4.978 |
