# Supplementary material for: The impact of access to financial services on mitigating COVID-19 mortality globally
Source: PLOS Glob Public Health. 2023 Mar 17;3(3):e0001137. doi: 10.1371/journal.pgph.0001137 (PMC10022804; doi:10.1371/journal.pgph.0001137)
Supplement: S2 Table — (DOCX) [file pgph.0001137.s002.docx]

**S2 Table. Summary Descriptive Statistics, Global Findex Variables**

| **Variable (% of population aged 15+)** | **Obs.** | **Mean** | **Std. Dev.** | **Min.** | **Max.** |
| --- | --- | --- | --- | --- | --- |
| Finance institution account | 142 | 0.585 | 0.294 | 0.086 | 0.999 |
| Borrowed from a financial institution or credit card | 142 | 0.240 | 0.187 | 0.024 | 0.828 |
| Saved at a financial institution | 142 | 0.238 | 0.197 | 0.016 | 0.793 |
| Debit card ownership | 142 | 0.443 | 0.314 | 0.017 | 0.988 |
| Credit card ownership | 142 | 0.193 | 0.210 | 0.001 | 0.826 |
| Received wages into financial institution | 142 | 0.246 | 0.199 | 0.016 | 0.683 |
| Paid utility bills using financial institution account | 142 | 0.231 | 0.240 | 0.000 | 0.813 |
| Outstanding housing loan | 142 | 0.130 | 0.108 | 0.011 | 0.543 |
| Used the internet for online transaction | 142 | 0.278 | 0.262 | 0.005 | 0.895 |
| Received wages through mobile phone | 142 | 0.025 | 0.028 | 0.000 | 0.134 |
| Paid utility bills through mobile phone | 142 | 0.068 | 0.074 | 0.000 | 0.371 |
| Made or received digital payments | 142 | 0.541 | 0.283 | 0.073 | 0.994 |
| Saved for old age | 142 | 0.210 | 0.155 | 0.024 | 0.614 |
| Borrowed for health or medical purposes | 142 | 0.100 | 0.066 | 0.006 | 0.311 |
| Coming up with emergency funds: possible | 142 | 0.554 | 0.165 | 0.165 | 0.927 |
| Main source of emergency funds: savings | 142 | 0.296 | 0.197 | 0.026 | 0.875 |
| Main source of emergency funds: formal loan | 142 | 0.059 | 0.044 | 0.002 | 0.243 |
| Borrowed from family or friends | 142 | 0.229 | 0.107 | 0.037 | 0.526 |
| Main source of emergency funds: family/friends | 142 | 0.317 | 0.155 | 0.052 | 0.711 |
| Main source of emergency funds: sale of assets | 142 | 0.042 | 0.055 | 0.000 | 0.281 |
