# Supplementary material for: The impact of access to financial services on mitigating COVID-19 mortality globally
Source: PLOS Glob Public Health. 2023 Mar 17;3(3):e0001137. doi: 10.1371/journal.pgph.0001137 (PMC10022804; doi:10.1371/journal.pgph.0001137)
Supplement: S3 Table — (DOCX) [file pgph.0001137.s003.docx]

**S3 Table. Correlation Matrix**

| **Variable** | ***ln(COVID-19 death rate per million, to 9/30/21)*** | ***Broad access to & use of formal finance index*** | ***Reliance on alternative, informal, & distress finance index*** | ***Population aged 65 & older, 2019 (%)*** | ***Population aged 0–14, 2019 (%)*** | ***ln(Population density per sq. mile, 2019)*** | ***Population in urban areas, 2018 (%)*** | ***ln(Per capita income, 2019)*** | ***Income inequality, 2019 (Gini)*** | ***ln(Mortality from indoor air pollution per 100K, 2016)*** | ***ln(Diabetes prevalence, %, 2019)*** | ***ln(Lung cancer prevalence per 100K, 2018)*** | ***Mean body mass index, 2016 (kg/m****^2^)* | ***Raised blood pressure prevalence, 2015 (%)*** | ***Tuberculosis vaccine coverage, 1989–2018 (%)*** | ***ln(Nurses & midwives per 10K, 2010–2019)*** | ***Health services effective coverage (UHC index), 2019*** |
| --- | --- | --- | --- | --- | --- | --- | --- | --- | --- | --- | --- | --- | --- | --- | --- | --- | --- |
| *ln(COVID-19 death rate per million, to 9/30/21)* | 1 |  |  |  |  |  |  |  |  |  |  |  |  |  |  |  |  |
| *Broad access to & use of formal finance index* | 0.31 | 1 |  |  |  |  |  |  |  |  |  |  |  |  |  |  |  |
| *Reliance on alternative, informal, & distress finance index* | -0.31 | -0.01 | 1 |  |  |  |  |  |  |  |  |  |  |  |  |  |  |
| *Population aged 65 & older, 2019 (%)* | 0.50 | 0.78 | -0.34 | 1 |  |  |  |  |  |  |  |  |  |  |  |  |  |
| *Population aged 0–14, 2019 (%)* | -0.60 | -0.75 | 0.40 | -0.85 | 1 |  |  |  |  |  |  |  |  |  |  |  |  |
| *ln(Population density per sq. mile, 2019)* | -0.02 | 0.18 | -0.26 | 0.18 | -0.28 | 1 |  |  |  |  |  |  |  |  |  |  |  |
| *Population in urban areas, 2018 (%)* | 0.45 | 0.64 | -0.19 | 0.49 | -0.61 | 0.02 | 1 |  |  |  |  |  |  |  |  |  |  |
| *ln(Per capita income, 2019)* | 0.58 | 0.84 | -0.23 | 0.75 | -0.88 | 0.17 | 0.77 | 1 |  |  |  |  |  |  |  |  |  |
| *Income inequality, 2019 (Gini)* | -0.15 | -0.38 | 0.29 | -0.44 | 0.44 | -0.15 | -0.14 | -0.38 | 1 |  |  |  |  |  |  |  |  |
| *ln(Mortality from indoor air pollution per 100K, 2016)* | -0.50 | -0.87 | 0.17 | -0.78 | 0.77 | -0.08 | -0.70 | -0.83 | 0.30 | 1 |  |  |  |  |  |  |  |
| *ln(Diabetes prevalence, %, 2019)* | 0.33 | 0.10 | -0.27 | 0.08 | -0.37 | 0.09 | 0.28 | 0.34 | -0.08 | -0.23 | 1 |  |  |  |  |  |  |
| *ln(Lung cancer prevalence per 100K, 2018)* | 0.62 | 0.64 | -0.38 | 0.78 | -0.86 | 0.14 | 0.52 | 0.78 | -0.48 | -0.68 | 0.35 | 1 |  |  |  |  |  |
| *Mean body mass index, 2016 (kg/m^2^)* | 0.60 | 0.38 | -0.25 | 0.38 | -0.55 | -0.13 | 0.67 | 0.64 | -0.21 | -0.55 | 0.48 | 0.53 | 1 |  |  |  |  |
| *Raised blood pressure prevalence, 2015 (%)* | -0.21 | -0.58 | 0.08 | -0.33 | 0.47 | -0.22 | -0.64 | -0.58 | 0.03 | 0.68 | -0.27 | -0.37 | -0.38 | 1 |  |  |  |
| *Tuberculosis vaccine coverage, 1989–2018 (%)* | -0.04 | -0.58 | 0.02 | -0.41 | 0.29 | -0.20 | -0.29 | -0.35 | 0.30 | 0.44 | 0.02 | -0.28 | -0.13 | 0.29 | 1 |  |  |
| *ln(Nurses & midwives per 10K, 2010–2019)* | 0.55 | 0.65 | -0.21 | 0.64 | -0.73 | 0.07 | 0.53 | 0.75 | -0.41 | -0.64 | 0.23 | 0.70 | 0.53 | -0.36 | -0.24 | 1 |  |
| *Health services effective coverage (UHC index), 2019* | 0.51 | 0.88 | -0.17 | 0.79 | -0.80 | 0.19 | 0.69 | 0.87 | -0.38 | -0.90 | 0.20 | 0.71 | 0.49 | -0.63 | -0.43 | 0.67 | 1 |
