# Supplementary material for: The impact of access to financial services on mitigating COVID-19 mortality globally
Source: PLOS Glob Public Health. 2023 Mar 17;3(3):e0001137. doi: 10.1371/journal.pgph.0001137 (PMC10022804; doi:10.1371/journal.pgph.0001137)
Supplement: S4 Table — (DOCX) [file pgph.0001137.s004.docx]

**S4 Table. Alternative Model Specifications**

| **Dependent variable** | **(1)** | **(2)** | **(3)** | **(4)** | **(5)** | **(6)** | **(7)** | **(8)** | | **(9)** | | **(10)** |
| --- | --- | --- | --- | --- | --- | --- | --- | --- | --- | --- | --- | --- |
| ln(COVID-19 death rate per million through 9-30-2021) | FIML-SEM  All Vars | OLS  All Vars | No Region Dummies | Fin Indexes  Only | Fin Indexes w/ Income | Demogr & Socio-Econ | Health Infrastr. | | Pop. Health & Infrastr. | | Post OLS remove p>.4 | FIML-SEM  Model 9 Vars |
| **Financial Access Index Variables** |  |  |  |  |  |  |  | |  | |  |  |
| *Broad access to & use* | -0.2990*** | -0.3160*** | -0.4099** | 0.1609 | -0.2655* | -0.3631** | -0.2934*** | | -0.3507** | | -0.3094*** | -0.2993*** |
| *of formal finance index* | (0.0591) | (0.0814) | (0.1212) | (0.1143) | (0.1138) | (0.1334) | (0.0725) | | (0.1230) | | (0.0639) | (0.0548) |
|  |  |  |  |  |  |  |  | |  | |  |  |
| *Reliance on alternative, informal,* | 0.1142*** | 0.0714 | 0.0107 | -0.4117** | -0.1351 | 0.0141 | -0.1624* | | 0.0223 | | 0.0829* | 0.1234*** |
| *& distress finance index* | (0.0232) | (0.0478) | (0.0628) | (0.1147) | (0.1135) | (0.0888) | (0.0756) | | (0.0739) | | (0.0345) | (0.0290) |
| **Demographic & Socioeconomic Variables** |  |  |  |  |  |  |  | |  | |  |  |
| *Population aged 65 & older (%)* | -0.0002 | 0.0039 | 0.0181 |  |  | 0.0865** |  | |  | |  |  |
|  | (0.0416) | (0.0457) | (0.0397) |  |  | (0.0278) |  | |  | |  |  |
|  |  |  |  |  |  |  |  | |  | |  |  |
| *Population aged 0–14 (%)* | -0.0405*** | -0.0456* | -0.0551** |  |  | -0.0659 |  | |  | | -0.0466** | -0.0322** |
|  | (0.0141) | (0.0221) | (0.0201) |  |  | (0.0347) |  | |  | | (0.0153) | (0.0152) |
|  |  |  |  |  |  |  |  | |  | |  |  |
| *ln(Population density per sq. mile)* | -0.0669 | -0.0452 | 0.0152 |  |  | -0.1812 |  | |  | |  |  |
|  | (0.0618) | (0.0687) | (0.0892) |  |  | (0.1136) |  | |  | |  |  |
|  |  |  |  |  |  |  |  | |  | |  |  |
| *Population in urban areas (%)* | 0.0034 | 0.0022 | 0.0038 |  |  | 0.0068 |  | |  | |  |  |
|  | (0.0078) | (0.0104) | (0.0092) |  |  | (0.0091) |  | |  | |  |  |
|  |  |  |  |  |  |  |  | |  | |  |  |
| *ln(Per capita income)* | 0.4763** | 0.4968 | 0.3080 |  | 1.5492*** | 0.9354*** |  | |  | | 0.5397** | 0.5002*** |
|  | (0.2242) | (0.2832) | (0.2346) |  | (0.2385) | (0.2219) |  | |  | | (0.1617) | (0.1587) |
|  |  |  |  |  |  |  |  | |  | |  |  |
| *Income inequality (Gini)* | 0.0501*** | 0.0579*** | 0.0620*** |  |  | 0.0238 |  | |  | | 0.0578*** | 0.0499*** |
|  | (0.0154) | (0.0096) | (0.0084) |  |  | (0.0289) |  | |  | | (0.0092) | (0.0168) |
| **Population Health Variables** |  |  |  |  |  |  |  | |  | |  |  |
| *ln(Mortality from indoor air* | 0.0596 | -0.0054 | -0.5089 |  |  |  |  | | -0.6715 | |  |  |
| *pollution per 100K)* | (0.3955) | (0.4110) | (0.4413) |  |  |  |  | | (0.3622) | |  |  |
|  |  |  |  |  |  |  |  | |  | |  |  |
| *ln(Diabetes prevalence, %)* | -0.4154* | -0.3411* | 0.0013 |  |  |  |  | | 0.1759 | | -0.4284*** | -0.4108** |
|  | (0.2200) | (0.1454) | (0.3167) |  |  |  |  | | (0.2872) | | (0.1152) | (0.1750) |
|  |  |  |  |  |  |  |  | |  | |  |  |
| *ln(Lung cancer prevalence per 100K)* | 0.6502** | 0.6658* | 0.2843 |  |  |  |  | | 0.5036 | | 0.6700** | 0.6647*** |
|  | (0.2742) | (0.3126) | (0.3296) |  |  |  |  | | (0.3149) | | (0.2520) | (0.2520) |
|  |  |  |  |  |  |  |  | |  | |  |  |
| *Mean body mass index* | -0.0350 | -0.0365 | 0.1648* |  |  |  |  | | 0.2254 | |  |  |
|  | (0.0433) | (0.0449) | (0.0804) |  |  |  |  | | (0.1168) | |  |  |
|  |  |  |  |  |  |  |  | |  | |  |  |
| *Raised blood pressure prevalence (%)* | 0.0908 | 0.0840 | 0.1052 |  |  |  |  | | 0.0852 | | 0.0900* | 0.0939*** |
|  | (0.0587) | (0.0699) | (0.0888) |  |  |  |  | | (0.0645) | | (0.0400) | (0.0358) |
|  |  |  |  |  |  |  |  | |  | |  |  |
| *Tuberculosis vaccine coverage (%)* | -0.0035 | -0.0053*** | -0.0084** |  |  |  |  | | -0.0024 | | -0.0050*** | -0.0030 |
|  | (0.0025) | (0.0014) | (0.0025) |  |  |  |  | | (0.0029) | | (0.0012) | (0.0027) |
| **Health Infrastructure Variables** |  |  |  |  |  |  |  | |  | |  |  |
| *ln(Nurses & midwives per 10K)* | 0.1988 | 0.2175 | 0.3173 |  |  |  | 0.6244** | | 0.3105 | | 0.1983 | 0.1975 |
|  | (0.1667) | (0.1815) | (0.2388) |  |  |  | (0.1872) | | (0.2614) | | (0.1641) | (0.1534) |
|  |  |  |  |  |  |  |  | |  | |  |  |
| *Health services effective coverage index* | 0.0424*** | 0.0382*** | 0.0336** |  |  |  | 0.0784*** | | 0.0467*** | | 0.0384*** | 0.0425*** |
|  | (0.0093) | (0.0094) | (0.0120) |  |  |  | (0.0165) | | (0.0126) | | (0.0042) | (0.0036) |
| **World Bank Region Dummy Variables** |  |  |  |  |  |  |  | |  | |  |  |
| *East Asia & Pacific* | -2.9121*** | -2.7222*** |  |  |  |  |  | |  | | -2.7276*** | -2.7887*** |
|  | (0.4156) | (0.4126) |  |  |  |  |  | |  | | (0.2219) | (0.1898) |
|  |  |  |  |  |  |  |  | |  | |  |  |
| *Europe & Central Asia* | -0.9212*** | -0.8984** |  |  |  |  |  | |  | | -0.9777** | -0.9412*** |
|  | (0.2648) | (0.3429) |  |  |  |  |  | |  | | (0.3203) | (0.2855) |
|  |  |  |  |  |  |  |  | |  | |  |  |
| *South Asia* | -0.3021 | -0.2916 |  |  |  |  |  | |  | | -0.3033 | -0.3653 |
|  | (0.2783) | (0.2575) |  |  |  |  |  | |  | | (0.3386) | (0.3914 |
|  |  |  |  |  |  |  |  | |  | |  |  |
| *Middle East & North Africa* | -0.5796*** | -0.5164** |  |  |  |  |  | |  | | -0.5724** | -0.6184** |
|  | (0.1709) | (0.1952) |  |  |  |  |  | |  | | (0.2037) | (0.2433) |
|  |  |  |  |  |  |  |  | |  | |  |  |
| *Sub-Saharan Africa* | -1.8408*** | -1.4714** |  |  |  |  |  | |  | | -1.5385** | -1.839** |
|  | (0.2977) | (0.5810) |  |  |  |  |  | |  | | (0.5503) | (0.4449) |
|  |  |  |  |  |  |  |  | |  | |  |  |
| *Constant* | -3.8209 | -3.7009 | -6.4935 | 5.7771*** | -8.9249*** | -2.7549 | -1.2151 | | -4.6518 | | -4.9776** | -4.9776*** |
|  | (2.8889) | (2.6583) | (3.3812) | (0.4472) | (2.2835) | (1.9304) | (0.6648) | | (3.3343) | | (1.8925) | (1.8925) |
| N | 142 | 132 | 132 | 142 | 141 | 138 | 139 | | 135 | | 135 | 142 |
| R^2^ | 0.7404 | 0.7288 | 0.6455 | 0.1859 | 0.4337 | 0.5265 | 0.4479 | | 0.5876 | | 0.7378 | 0.7367 |
| Ramsey RESET Test  Prob > F |  | F=1.73  p=0.165 | F=1.22  p=0.306 | F=5.89***  p=0.001 | F=4.38***  p=0.006 | F=0.55  p=0.648 | F=5.24***  p=0.002 | | F=1.49  p=0.221 | | F=1.70  p=0.171 |  |
| Link Test  Prob > t (prediction^2^ coef.) |  | p=0.123 | p=0.345 | p = 0.069* | p = 0.491 | p=0.478 | p=0.525 | | p=0.545 | | p=0.128 |  |
| Wald test on added variables (vs. Model #)  F  Prob > F |  | (vs. 3)  F=534***  p=0.000 | (vs. 8)  F=105***  p=0.000 | base | (vs. 4)  F=42.2***  p=0.001 | (vs. 5)  F=31.9***  p=0.000 | (vs. 4)  F=94.4***  p=0.000 | | (vs. 7)  F=383***  p=0.000 | | (2 vs. 9)  F=1.31  p=0.372 |  |

*Notes*: robust standard errors in parentheses; two-tailed significance levels ***0.01, **0.05, *0.10

Alternative Specifications

Model 1: Full Information Maximum Likelihood Structural Equation Model, missing values method, all variables, including regional dummies; main model, repeated from Table 5

Model 2: OLS, all variables, including regional dummies

Model 3: OLS, all variables except no regional dummies

Model 4: OLS, only financial access indexes

Model 5: OLS, only the financial access indexes together with per capita income

Model 6: OLS, financial access indexes with only demographic and socioeconomic variables

Model 7: OLS, only financial access indexes with health infrastructure variables (which are strongly correlated with per capita income)

Model 8: OLS, financial access variables with only the health-related variables

Model 9: OLS, post OLS parsimony model, removes from Model 1 all variables with p<0.40

Model 10: Full Information Maximum Likelihood Structural Equation Model, missing values method, using variables in parsimony Model 9
