# Supplementary material for: The impact of access to financial services on mitigating COVID-19 mortality globally
Source: PLOS Glob Public Health. 2023 Mar 17;3(3):e0001137. doi: 10.1371/journal.pgph.0001137 (PMC10022804; doi:10.1371/journal.pgph.0001137)
Supplement: S5 Table — (DOCX) [file pgph.0001137.s005.docx]

**S5 Table. Models Including COVID-19 Vaccination Variables**

| **Dependent variable** | **(1)** | **(2)** | **(11)** | **(12)** | **(13)** | **(14)** |
| --- | --- | --- | --- | --- | --- | --- |
| ln(COVID-19 death rate per million through 9-30-2021) | FIML-SEM  All Vars | OLS  All Vars | OLS  w/ Vaccine Rate | OLS  w/ Vaccine  Availability | FIML-SEM  Model 11  Vars | FIML-SEM  Model 12  Vars |
| **Financial Access Index Variables** |  |  |  |  |  |  |
| *Broad access to & use* | -0.2990*** | -0.3160*** | -0.2930*** | -0.2872** | -0.2808*** | -0.2903*** |
| *of formal finance index* | (0.0591) | (0.0814) | (0.0776) | (0.0916) | (0.0594) | (0.0591) |
|  |  |  |  |  |  |  |
| *Reliance on alternative, informal,* | 0.1142*** | 0.0714 | 0.0829 | -0.0036 | 0.1234*** | 0.1137*** |
| *& distress finance index* | (0.0232) | (0.0478) | (0.0560) | (0.1856) | (0.0271) | (0.0338) |
| **Demographic & Socioeconomic Variables** |  |  |  |  |  |  |
| *Population aged 65 & older (%)* | -0.0002 | 0.0039 | 0.0038 | -0.0063 | -0.0009 | -0.0071 |
|  | (0.0416) | (0.0457) | (0.0459) | (0.0614) | (0.0416) | (0.0442) |
|  |  |  |  |  |  |  |
| *Population aged 0–14 (%)* | -0.0405*** | -0.0456* | -0.0471* | -0.0182 | -0.0418*** | -0.0464*** |
|  | (0.0141) | (0.0221) | (0.0229) | (0.0111) | (0.0155) | (0.0175) |
|  |  |  |  |  |  |  |
| *ln(Population density per sq. mile)* | -0.0669 | -0.0452 | -0.0325 | -0.1238 | -0.0577 | -0.0212 |
|  | (0.0618) | (0.0687) | (0.0741) | (0.1184) | (0.0635) | (0.0546) |
|  |  |  |  |  |  |  |
| *Population in urban areas (%)* | 0.0034 | 0.0022 | 0.0028 | -0.0002 | 0.0037 | 0.0047 |
|  | (0.0078) | (0.0104) | (0.0101) | (0.0150) | (0.0073) | (0.0081) |
|  |  |  |  |  |  |  |
| *ln(Per capita income)* | 0.4763** | 0.4968 | 0.5508 | 0.6335 | 0.5245** | 0.5635*** |
|  | (0.2242) | (0.2832) | (0.2856) | (0.5532) | (0.2071) | (0.2144) |
|  |  |  |  |  |  |  |
| *Income inequality (Gini)* | 0.0501*** | 0.0579*** | 0.0566*** | 0.0415*** | 0.0489*** | 0.0492*** |
|  | (0.0154) | (0.0096) | (0.0098) | (0.0112) | (0.0155) | (0.0148) |
| **Population Health Variables** |  |  |  |  |  |  |
| *ln(Mortality from indoor air* | 0.0596 | -0.0054 | -0.0030 | 0.3002 | 0.0540 | 0.1355 |
| *pollution per 100K)* | (0.3955) | (0.4110) | (0.3433) | (0.3712) | (0.3326) | (0.2486) |
|  |  |  |  |  |  |  |
| *ln(Diabetes prevalence, %)* | -0.4154* | -0.3411* | -0.3433* | 0.3896 | -0.4123* | -0.4142* |
|  | (0.2200) | (0.1454) | (0.1484) | (0.2393) | (0.2211) | (0.2269) |
|  |  |  |  |  |  |  |
| *ln(Lung cancer prevalence per 100K)* | 0.6502** | 0.6658* | 0.6861* | 0.5001 | 0.6759*** | 0.6589*** |
|  | (0.2742) | (0.3126) | (0.2835) | (0.2811) | (0.2460) | (0.2540) |
|  |  |  |  |  |  |  |
| *Mean body mass index* | -0.0350 | -0.0365 | -0.0551 | -0.2031 | -0.0532 | -0.0639 |
|  | (0.0433) | (0.0449) | (0.0527) | (0.1056) | (0.0487) | (0.0592) |
|  |  |  |  |  |  |  |
| *Raised blood pressure prevalence (%)* | 0.0908 | 0.0840 | 0.0852 | 0.0912 | 0.0930 | 0.1074 |
|  | (0.0587) | (0.0699) | (0.0698) | (0.1141) | (0.0590) | (0.0673) |
|  |  |  |  |  |  |  |
| *Tuberculosis vaccine coverage (%)* | -0.0035 | -0.0053*** | -0.0051*** | -0.0070** | -0.0034 | -0.0027 |
|  | (0.0025) | (0.0014) | (0.0011) | (0.0020) | (0.0024) | (0.0031) |
| **Health Infrastructure Variables** |  |  |  |  |  |  |
| *ln(Nurses & midwives per 10K)* | 0.1988 | 0.2175 | 0.2095 | 0.2657 | 0.1905 | 0.1534 |
|  | (0.1667) | (0.1815) | (0.1785) | (0.2348) | (0.1630) | (0.1376) |
|  |  |  |  |  |  |  |
| *Health services effective coverage index* | 0.0424*** | 0.0382*** | 0.0367** | 0.0403* | 0.0407*** | 0.0422*** |
|  | (0.0093) | (0.0094) | (0.0101) | (0.0170) | (0.0096) | (0.0086) |
| **World Bank Region Dummy Variables** |  |  |  |  |  |  |
| *East Asia & Pacific* | -2.9121*** | -2.7222*** | -2.8721*** | -3.0250*** | -3.0534*** | -3.0944*** |
|  | (0.4156) | (0.4126) | (0.2773) | (0.6080) | (0.2850) | (0.2807) |
|  |  |  |  |  |  |  |
| *Europe & Central Asia* | -0.9212*** | -0.8984** | -0.9803** | -1.0510 | -1.0006*** | -0.9206*** |
|  | (0.2648) | (0.3429) | (0.2903) | (0.7638) | (0.2037) | (0.2240) |
|  |  |  |  |  |  |  |
| *South Asia* | -0.3021 | -0.2916 | -0.4296 | -2.0846** | -0.4322 | -0.6104 |
|  | (0.2783) | (0.2575) | (0.2648) | (0.6747) | (0.2906) | (0.4203) |
|  |  |  |  |  |  |  |
| *Middle East & North Africa* | -0.5796*** | -0.5164** | -0.5438** | -1.0684** | -0.6023*** | -0.5973*** |
|  | (0.1709) | (0.1952) | (0.2191) | (0.3658) | (0.1919) | (0.1784) |
|  |  |  |  |  |  |  |
| *Sub-Saharan Africa* | -1.8408*** | -1.4714** | -1.5827* | -2.2056* | -1.9268*** | -1.9679*** |
|  | (0.2977) | (0.5810) | (0.6813) | (1.0031) | (0.3881) | (0.4406) |
| **COVID-19 Vaccine Status, June 2021** |  |  |  |  |  |  |
| *Vaccination Rate (share of population)* |  |  | -0.7047** | -1.2119 | -0.5523* | -0.6378** |
|  |  |  | (0.2644) | (0.7312) | (0.3099) | (0.3102) |
|  |  |  |  |  |  |  |
| *Availability (full does available per capita)* |  |  |  | 0.1631 |  | 0.2093 |
|  |  |  |  | (0.1940) |  | (0.2135) |
|  |  |  |  |  |  |  |
| *Constant* | -3.8209 | -3.7009 | -3.4694 | -2.1244 | -3.5553 | -4.4687* |
|  | (2.8889) | (2.6583) | (2.7169) | (7.0472) | (2.7455) | (2.4504) |
|  |  |  |  |  |  |  |
| N | 142 | 132 | 132 | 77 | 142 | 142 |
| R^2^ | 0.7404 | 0.7288 | 0.7310 | 0.6710 | 0.7423 | 0.7453 |
| Ramsey RESET Test  Prob > F |  | F=1.73  p=0.165 | F=1.43  p=0.240 | F=3.05  p=0.037 |  |  |
| Link Test  Prob > t (prediction^2^ coef.) |  | p=0.123 | p=0.177 | p=0.030 |  |  |

*Notes*: robust standard errors in parentheses; two-tailed significance levels ***0.01, **0.05, *0.10;
vaccination rate data source [57]; vaccination availability data source [58]

Alternative Model Specifications

Model 1: Full Information Maximum Likelihood Structural Equation Model, missing values method; main model, repeated from Table 5

Model 2: OLS, all main model variables, including regional dummies, repeated from S4 Table

Model 11: OLS, Model 2 variables plus COVID-19 vaccination rate

Model 12: OLS, Model 2 variables plus COVID-19 vaccination rate and vaccine availability

Model 13: Full Information Maximum Likelihood Structural Equation Model, missing values method, using variables in Model 11

Model 14: Full Information Maximum Likelihood Structural Equation Model, missing values method, using variables in Model 12
