# Supplementary figures and images for: The impact of access to financial services on mitigating COVID-19 mortality globally
Source: PLOS Glob Public Health. 2023 Mar 17;3(3):e0001137. doi: 10.1371/journal.pgph.0001137 (PMC10022804; doi:10.1371/journal.pgph.0001137)

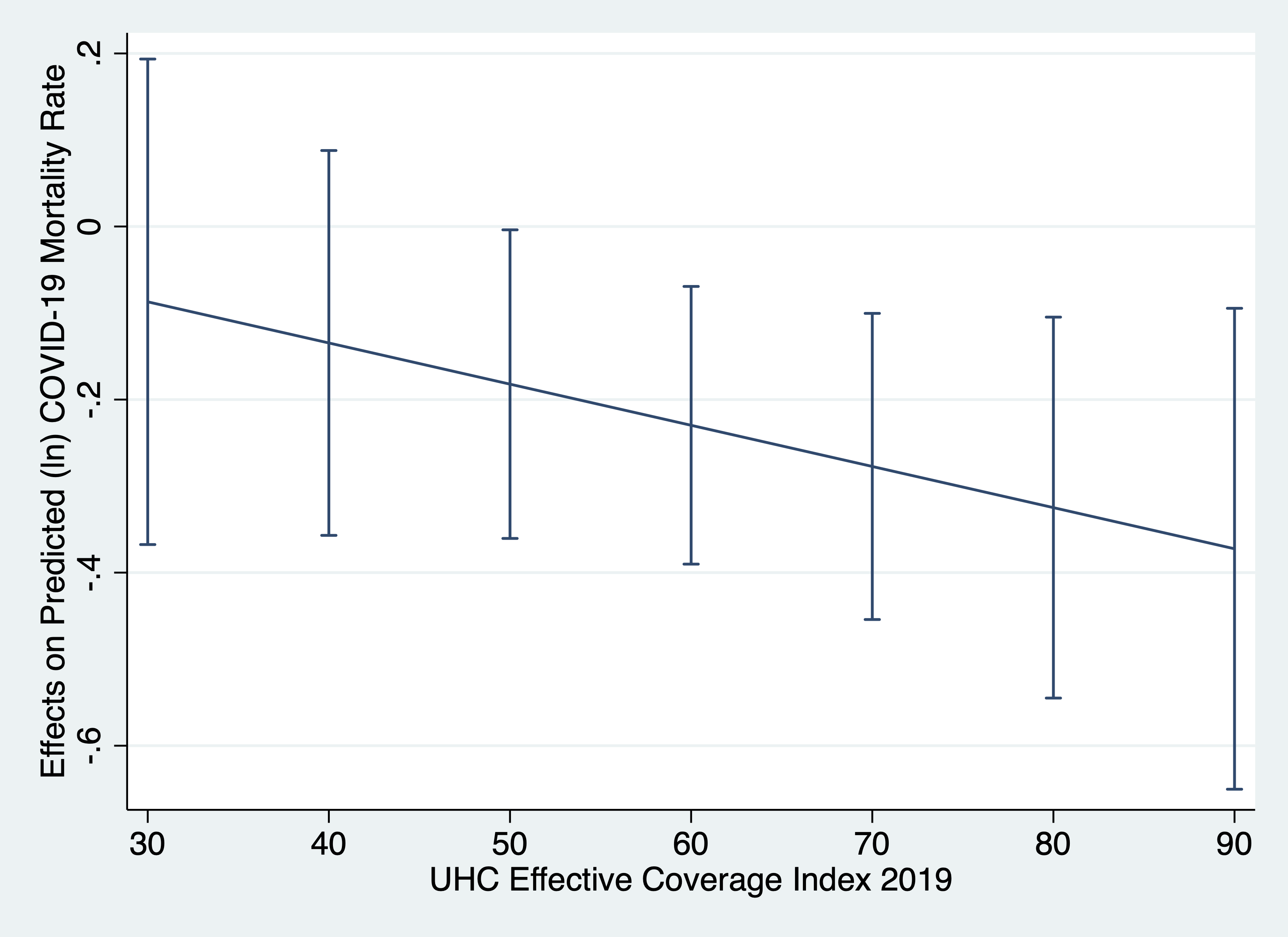

Supplement: S1 Fig — (TIFF) [file pgph.0001137.s006.tiff]

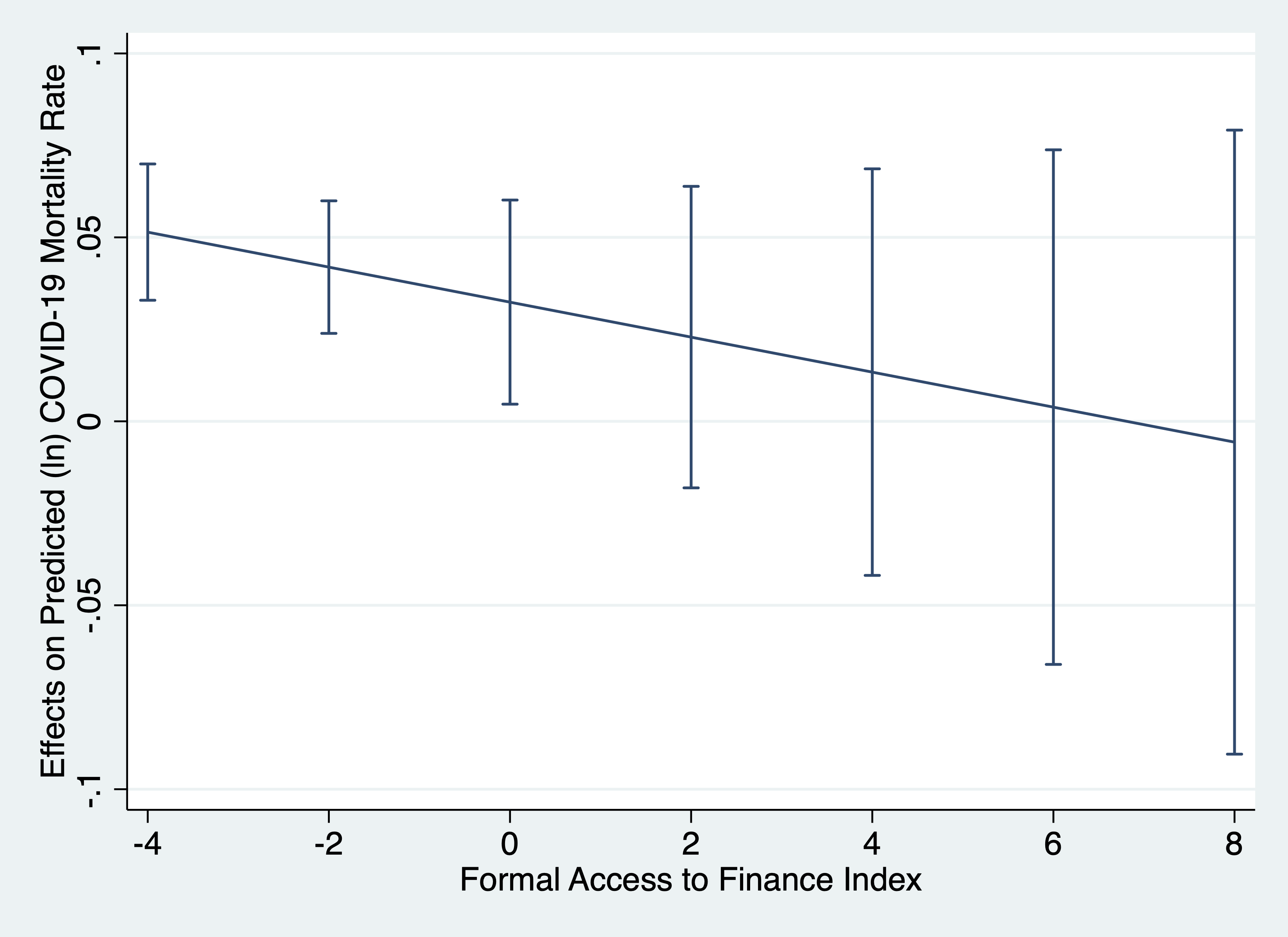

Supplement: S2 Fig — (TIFF) [file pgph.0001137.s007.tiff]
